# Understanding of and adherence to advice after telephone counselling by nurse: a survey among callers to a primary emergency out-of-hours service in Norway

**DOI:** 10.1186/1757-7241-19-48

**Published:** 2011-09-05

**Authors:** Elisabeth Holm Hansen, Steinar Hunskaar

**Affiliations:** 1 National Centre for Emergency Primary Health Care, Uni Health, Kalfarveien 31, NO-5018 Bergen, Norway; 2 Research Group for General Practice, Department of Public Health and Primary Health Care, University of Bergen, Kalfarveien 31, NO-5018 Bergen, Norway

**Keywords:** triage, self-care advice, counselling by nurse, out-of-hours services

## Abstract

**Background:**

To investigate how callers understand the information given by telephone by registered nurses in a casualty clinic, to what degree the advice was followed, and the final outcome of the condition for the patients.

**Methods:**

The study was conducted at a large out-of-hours inter-municipality casualty clinic in Norway during April and May 2010. Telephone interviews were performed with 100 callers/patients who had received information and advice by a nurse as a sole response. Six topics from the interview guide were compared with the telephone record files to check whether the caller had understood the advice. In addition, questions were asked about how the caller followed the advice provided and the patient's outcome.

**Results:**

99 out of 100 interviewed callers stated that they had understood the nurse's advice, but interpreted from the telephone records, the total agreement for all six topics was 82.6%. 93 callers/patients stated that they followed the advice and 11 re-contacted the casualty clinic. 22 contacted their GP for the same complaints the same week, of whom five patients received medical treatment and one was hospitalised. There were significant difference between the native-Norwegian and the non-native Norwegian regarding whether they trusted the nurse (p = 0.017), and if they got relevant answers to their questions (p = 0.005).

**Conclusion:**

Callers to the out-of-hours service seem to understand the advice given by the registered nurses, and a large majority of the patients did not contact their GP or other health services again with the same complaints.

**Practice Implication:**

Medical and communicative training must be an important part of the continuous improvement strategy within the out-of-hour services.

## 1. Introduction

Telephone consultation and triage by nurses constitute an important and central part of the out-of-hours services in several countries [1-7]. The consultation may be completed with medical advice given by the nurse as the sole response, or may result in a referral to another level of care if appropriate. Several studies have investigated the quality and safeness of this kind of service, and also the outcome after the nurse's advice and triage. Some previous studies indicate that advice given by nurses only delay consultation by a general practitioner [GP], while other studies claim to show that nurse advice reduce the GP's workload [8-14]. Several papers state that patients generally have a good understanding of the advice given, but very few compare the patient's answers with a telephone record file [13,15-20].

In Norway three quarters of all contacts to casualty clinics are assessed as non-urgent [21], which means that a lot of the contacts could be handled through self-care or a visit to a GP the following day. About one fourth of the contacts to the out-of-hours services in Norway are managed by nurses giving medical advice [21], but no one has investigated the content of this service. All medical advice by nurses in Norwegian casualty clinics is recorded in electronic medical files, and in many casualty clinics all telephone conversations are also tape recorded and stored.

In this study we have investigated how callers understand the medical information and advice given to them by nurses in a casualty clinic. We have compared the information extracted from the telephone record file with information obtained by telephone interviews with the callers some days later. In addition, we have investigated to what degree the patients followed the advice given, and the consequences of the advice.

## 2. Methods

### 2.1 Sample

The study was conducted at a large out-of-hours inter-municipality casualty clinic in Norway during April and May 2010. One hundred callers/patients were interviewed about their telephone consultation with a nurse on average nine days afterwards. The casualty clinic serves four municipalities with more than 100 000 inhabitants, and the patients can call directly to the clinic. The casualty clinic is staffed with doctors and nurses all day throughout the week.

During 2009 about 59 000 contacts were received at the casualty clinic by telephone and direct attendance, and 27% of the contacts were handled by registered nurses [RN] as a sole response (personal communication). A total of 28 RNs were employed at the casualty clinic and their tasks were to receive calls from patients, their families, or others, to assess the priority grade and decide on different possible actions by giving self-care advice or referring to another appropriate level of care. The latter could be a medical consultation by a doctor, a home visit or sending an ambulance. All telephone calls to the casualty clinic were recorded. The nurses who operated the telephones also met the patients face to face if the latter attended the clinic to see a medical doctor.

Information about the study was given to the nurses at two staff meetings, first with the head nurse and medical director and then by the researcher and head nurse. The RNs who worked in the casualty clinic agreed to participate in the study, and all nurses consented to using their telephone record logs. They were not informed about how the callers were to be recruited to the study.

### 2.2 Recruitment

The decision to include until 100 callers had conducted an interview was based on a trade-off between resources and an acceptable sample size. The former includes the total capacity of the staff at the actual clinic and the time available for the researcher and the research assistant; the latter comprised a subjective appraisal of the gain in precision (width of a confidence interval) obtained by increasing the sample size in the range from 50 to 200.

In order to obtain a representative sample and avoid bias, we used a recruitment strategy where two callers, the first and the last, who had received medical advice by nurse as a sole response during daytime [08.00-15.30], afternoon [15.30-22.30] and night shift [22.30-08.00], were chosen. The consultations concerned the callers themselves or someone in the callers' families, for example a child.

The head nurse served as a research assistant, and her tasks were to identify and contact the callers, inform about the study and invite them to participate. During the contact she made an appointment for a telephone interview with the researcher. If a patient did not want to participate in the study the next/former caller [depending on whether it was the first/last at the shift] was invited. After the information was given by phone, a letter of information including a consent form was sent to each caller/patient together with a return envelope. A list with ID, name, telephone number and time and day of appointment for each person recruited was sent to the researcher who carried out the interview.

### 2.3 Information from the telephone records

The research assistant listened to the telephone records and collected data on the reasons for contacting the casualty clinic. Age and gender of the caller and patient were registered, and the following six questions regarding the consultation, were answered as "Yes", "Partly", "No" or "Not relevant". Further details were written down and compared to the information gathered in the interview:

[1] Did the caller get enough time to explain his or her complaints? This was an assessment made by the research assistant.

[2] Did the caller get understandable medical advice from the nurse? Specific advice was written down.

[3] Did the caller get understandable information about what to look for? If the caller was told to look for something this was written down.

[4] Did the caller get the option to call back, if necessary? If the caller received such information the time schedule was written down.

[5] Did the caller get information on why a patient could wait and see in that particular situation? If relevant, the reason for why they could wait and see was written down.

[6] Did the caller get information on if or when to contact their GP during daytime? If relevant, the time schedule was written down.

Due to Norwegian regulations, the researcher was not allowed to have access to the telephone records. Before the first telephone interview the research assistant and the researcher together listened to four anonymous telephone record files and filled out the questionnaire in order to reduce variability in the interpretation of the counselling.

### 2.4 Interviews with callers/patients

An interview form was developed, where the six questions from the telephone record form were included and classified in the same way as was done in the telephone records. ("Yes", "Partly", "No" or "Not relevant"). Additional details were written down and compared to the information gathered in the telephone records). Further, the callers were asked if they generally understood the information and medical advice communicated by the nurse, if the caller/patient followed the advice given and the outcome of the condition. In addition they were asked if they trusted the nurse, if they got worse or better after the contact, if they contacted their GP or re-contacted the casualty clinic. They were also asked if they had rather wanted to see a doctor. If they contacted the GP or casualty clinic, they were asked if they got any treatment and what kind of treatment. Patients referred to hospital, were asked about the medical treatment received. The answers were registered in the same categories as the six questions which were compared to the telephone record file. The researcher was blinded for all the information from the telephone record forms when the interviews were carried out.

### 2.5 Data analysis

SPSS version 15.0 and STATA version 11.0 was used to analyse data. The analyses in this study comprise two parts. Firstly, the six variables concerning the counselling are evaluated for agreement, reported both as actual agreement and as Cohen's kappa.

Three main outcome variables; whether the given advice was followed and if a GP-contact or a re-contact to the casualty clinic took place, were analysed for associations with some potential predictive variables. Exact methods, Fischer's test and logistic regression, were all used due to several occurrences of small and zero-cells in cross tabulations.

The study was approved by the Privacy Ombudsman for Research.

## 3. Results

A total of 134 callers were contacted by the research assistant at the recruitment stage. Fifteen persons [11%] could not participate in the study for various reasons; eight persons [6%] did not want to participate; four callers [3%] were on travel abroad; one had exams; one caller was in hospital, and one caller had a bad telephone line. 19 callers had not answered the telephone from the researcher after three attempts. These 19 callers were not significantly different from the participating callers/patients regarding age, gender, number of days between advice and interview, time of day or duration of calls.

One hundred callers/patients were interviewed about their telephone consultation with an RN at the casualty clinic. Callers mean age was 37 years [range 19-83 years] and mean age of patients was 18 years [range 0-72 years]. Most callers were women [55%], and mean number of days between call and interview was 9 days [range 2-14 days]. 24% were interviewed within 7 days and 93% within 11 days. The distribution of the calls during the day was 37% in daytime, 42% in the afternoon and 21% at night. There were no significant differences between responders and non-responders regarding these variables.

Mean length of the 100 calls was 4 min and 1 s [range 1-12 min]. Telephone calls regarding psychiatric problems had the longest durations. There were no significant differences among responders and non-responders regarding caller's age or gender, regarding the patient's age or gender, time of day, duration of calls and/or days between the counselling and interview.

Among the 100 calls the most frequent reasons for contact were fever (23%), vomiting/diarrhoea (10%), abdominal pain (9%), question about drugs (9%), skin problems (9%), ear ache (6%) and others (34%). 88% of the 67 callers who contacted the casualty clinic on behalf of someone other than themselves called on behalf of their children under 16 years of age.

Table 1 shows the answers to the six questions from the 100 callers written down from the telephone record, and the answers to the same questions from the interviews. The categories of answers to the six questions were: "yes", "no", "partly" or "not-relevant. The observed agreement and kappa values are also presented in Table 1. Before the analyses of agreement and kappa, the category "not-relevant" was re-classified to "no" when both research assistant and caller had registered "not-relevant" or when one of them had answered "not-relevant" and the other had answered "no". Similarly the category "not-relevant" was re-classified to "yes" when one answered "yes" and the other answered "not-relevant".

**Table 1:** The six variables concerning the counselling as interpreted from the telephone record and reported by the callers are evaluated for agreement, reported both as actual agreement and as Cohen's kappa

	Telephone record				Caller/Patient				Observed agreement*	Cohen's kappa*
	**Yes**	**Partly**	**No**	**Not relevant**	**Yes**	**Partly**	**No**	**Not relevant**		
Did caller get enough time to explain her/his complaints?	100	0	0	0	94	3	3	0	94	NA
Did caller get understandable medical advice from the nurse?	74	6	6	14	78	9	5	8	82	0.39
Did caller get understandable information about what to look for?	60	7	14	19	68	4	19	9	73	0.38
Did caller get the option to call back, if necessary?	63	2	25	10	79	2	9	10	77	0.42
Did caller get information on why a patient could wait and see in that particular situation?	65	10	6	19	74	4	12	10	76	0.32
Did caller get information on if or when to contact their GP during daytime?	33	1	48	18	31	1	43	25	82	0.63

*When Observed agreement and Cohen's kappa were analysed, "not relevant" was recoded to either "no" or "yes". The category "not-relevant" was re-classified to "no" when both research assistant and caller had registered "not-relevant" or when one of them had answered "not-relevant" and the other had answered "no". Similarly the category "not-relevant" was re-classified to "yes" when one answered "yes" and the other answered "not-relevant".

In the interview a question regarding of the overall understanding during the conversation with the nurse was posed, and all except one caller said that they understood the information and medical advice given. When comparing the answers with the telephone record the observed agreement was 82.6%.

Table 2 presents the outcomes of the telephone consultations as reported in the interviews for the variables "Followed the advice", "Contacted GP" and "Re-contacted casualty clinic". The analyses included the following independent variables: Gender, native Norwegian/others, time of day for consultation, whether the condition got worse after the contact with the nurse, and information concerning how the caller/patient experienced the telephone consultations with respect to whether they had enough time, received relevant answers to questions and whether they trusted the nurse. All men and 91% of the women stated that they followed the advice (p = 0.34 for gender difference). The variables time of day of the call, whether the caller got answer to the questions and trusted the nurse were significant predictors for following the advice.

**Table 2:** Outcome after nurse's telephone advice, by gender and origin of caller and some characteristics regarding the consultation

	All	Followed the advices			Contacted GP			Re-contact Casualty clinic		
	N = 100	Yes N = 93	No N = 7	p-value	Yes N = 22	No N = 78	p-value	Yes N = 11	No N = 89	p-value
Origin of caller				0.08			> 0.99			> 0.99
Native Norwegian	84	80	4		19	65		10	74	
Others	16	13	3		3	13		1	15	
Gender of caller				0.34			> 0.99			0.07
Men	22	22	0		5	17		5	17	
Women	78	71	7		17	61		6	72	
Time of day				0.009			0.47			> 0.99
Daytime	37	34	3		9	28		4	33	
Afternoon	42	42	0		7	35		5	37	
Night	21	17	4		6	15		2	19	
Got enough time				0.06			0.39			> 0.99
Yes	94	89	5		20	74		11	83	
No	3	2	1		1	2		0	3	
Partly	3	2	1		1	2		0	3	
Got worse				0.53			0.039			0.012
Yes	10	9	1		5	5		4	6	
No	90	84	6		17	73		7	83	
Got answers to the questions				< 0.0001			0.024			
Yes	79	79	0		13	66		10	69	> 0.99
No	6	3	3		2	4		0	6	
Partly	15	11	4		7	8		1	14	
Trusted the nurse				< 0.0001			0.32			0.64
Yes	74	74	0		14	60		10	64	
No	8	6	2		3	5		0	8	
Partly	18	13	5		5	13		1	17	

Due to zero-cells a full multivariable analysis was impossible, but some pragmatic partial models could be explored. None of the other independent variables influenced the association with time of day of the call. This was also the case for the highly significant relations between following advice and getting answers to questions and trusting the nurse, but the two could not be analysed in the same model, again due to zero-cells. As is shown in table 2 everyone who got answers to their questions and also those who trusted the nurse followed the advice. Of the 100 callers, 22 contacted a GP afterwards, and this was significantly associated with the patient getting worse after the consultation. Re-contact to the casualty clinic was also associated with experiencing deterioration of the clinical symptoms.

The age of the callers, whether the callers were told what to look for, and why it was not necessary to see a doctor at that time, did not have statistically significant relations to any of the three dependent variables in table 2.

Callers who did speak fluent Norwegian and had Norwegian names were compared to callers who did not speak fluent Norwegian and had foreign names. There were significant differences between the two groups regarding whether they trusted the nurse (p = 0.017). Furthermore there were differences between the two group regarding comprehension of the medical advice and whether they followed them, but these differences did not reach significance.

Only 23% of the callers contacted health personnel for the same problem after the advice given by the nurse. Actually 13 [36%] of the 36 callers who stated that they were told when or whether to contact their GP next day did so, and of the 62 who stated that they were not told to do so, 9 [14.5%] in fact did [p = 0.03]. Five of the 100 callers/patients stated that they would prefer to talk to a doctor instead of the nurse on the phone. All five callers who would prefer talking to a doctor reported following the advice given by the nurse. The length of the telephone consultation or the type of complaint did not affect whether they followed the nurse's advice.

Among the eight callers who answered that they did not trust the nurse, one would rather prefer talking to a doctor. As for the 18 callers who answered that they partly trusted the nurse three would prefer a doctor. Among the callers who told that they would prefer a doctor two persons contacted their GP and none contacted the casualty clinic.

**Figure 1:**
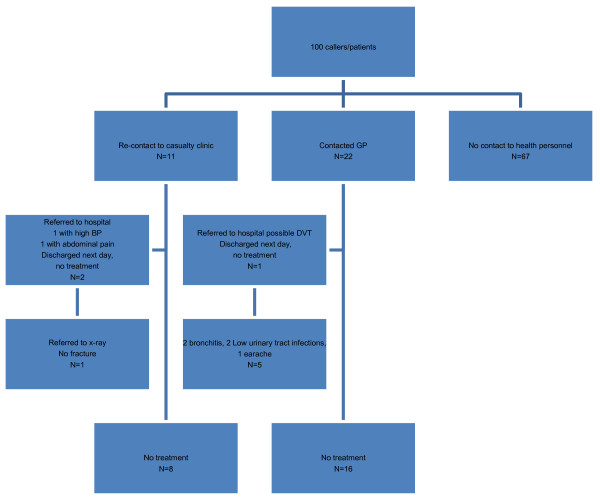
**Follow-up for all 100 callers/patients who received advice from a nurse**.

In the interview 79% stated that they got relevant answers to their questions, 15% did partly get relevant answers, while 6% did not get relevant answers. There were significant differences among the native-Norwegian and the non-native group, where 25% answered that they did not get relevant answers to their question in the non-native group, while in the native-Norwegian group the corresponding figure was only 2% (p = 0.005). Figure 1 shows a follow-up chart for some more details for all callers/patient's history.

## 4. Discussion and conclusion

### 4.1 Discussion

This is the first study in Norway investigating caller's adherence to and outcomes of telephone counselling by nurses in out-of-hours primary care emergency services. Most of the callers/patients stated that they understood and followed the advice, and the observed agreement found between telephone records and interviews were satisfactory even with a disagreement of 18%. Most callers did not re-contact health personnel regarding the same complaints during the following week.

Several studies have investigated whether patients followed the advice given by a nurse. However, we found few studies that reported the use of actual telephone records to compare advice given by nurses against advice reported by caller in interviews. The use of telephone contacts in our study was in accordance with studies from US, Australia, New Zealand and Sweden [6,8,16,22-24]. Parents calling on behalf of young children and the fact that women contacted the casualty clinic more often than men were also typical in other studies [15,16,22,24].

Almost everybody stated that they understood the RN's medical advice on how to deal with the conditions, but there were some discrepancies when comparing the reported advice in the interviews against the record files. This corresponds to the studies from Dale et al., and Leclerc et al. [17,19]. One way to ensure that the information is understood is to ask the caller to repeat the advices given by the nurse at the end of the telephone call, but this intervention has received little attention in studies in which nurse advice has been discussed.

A rather high proportion followed the nurse's advices in our study compared to former studies from US, UK and Canada [16,17,20,22,24,25], and a much lower proportion of patients re-contacted the GP. In our study we have interviewed patients/callers several days later. Thus we have a much longer follow-up period than most of the other studies we found on this topic. One study from the Netherlands [9] stated that almost half of the patients in the study who contacted the GP cooperative attended their own GP during office hours within a week. These patients had been seeing a doctor but there were still a very high proportion of contacts to the patient's own GP.

The fact that the non-Norwegian group trusted the nurse to a lesser extent than the native-Norwegian group, and did not get relevant answers to the same degree, is an important result. If the caller's language skills are limited it is of utmost importance that nurses articulate themselves clearly, avoid unnecessary or difficult words, and ask the caller so repeat the advice. Nurses should perhaps spend more time ensuring that the callers have understood the information. It must be remarked that the non-Norwegian group was not hard to understand during the interviews, and there were only minor difficulties when asking the questions.

A definite strength of our study is that we in fact compared the answers from the callers/patients by listening to telephone record files. We were also able to follow the patients until several days after the telephone contacts to check the patient outcome. Possible compliance, and callers eager to please the researcher during the interviews could constitute a weakness. We therefore stated in every interview that the researcher had no work connection to or affiliation with the casualty clinic, and that every caller/patient was ensured anonymity. It must be mentioned that the nurses might have changed their usual behaviour on the telephone, such as being more kind or pleasant at the start of the study. On the other hand the nurses did not know which telephone records we selected, and their medical skills could not have been improved during the short time of the study. Memory bias regarding the issues raised in the interviews could be a possible limitation, but when comparing the answers from callers/patients with the record file we found identical wording in most of the cases. Only two persons stated that they were unsure whether they were told if or when to contact their GP.

Even when callers answered that they did not feel quite confident regarding the advice, they followed them. This raises the question of whether nurses wield authority in a potentially dangerous way that might influence the callers. Nurses need to be aware of the caller's vulnerability and try to build a relationship of trust quite early in the conversation [26]. Nurses who provide telephone advice and counselling must also be aware that they have a duty to and responsibility for the caller/patient. It is also of outmost importance that the nurses possess the relevant and adequate information to provide correct advice. Good medical knowledge and communication skills are necessary to meet the callers' needs, and callers'/patients' levels of knowledge vary [27-29]. These days many patients have been reading about the medical condition on the Internet before they contact the casualty clinic. This challenges the nurse's knowledge and skills, and nurses in casualty clinics should have a profound medical knowledge and a good experience base. Continuous training to improve both medical knowledge and communication skills should be carried out in all casualty clinics and telephone call centres. In addition, casualty clinics should have a policy communicated to the inhabitants to ensure that they have the relevant expectation to the service.

### 4.2 Conclusion

Nurse telephone consultations and counselling constitute an independent service in which callers have high expectations. A high share of the callers understood the advice and followed them. Two thirds of the callers who received advice from nurses had no contact with their GP, casualty clinic or other health personnel the following week. Non-Norwegian callers challenge the nurse's communicative skills both through language and cultural backgrounds.

### 4.3 Practice implication

Nurses who give self-care advice must ensure that callers are able to handle this responsibility. One way to ensure that the self-care advice is understood could be to ask the callers to repeat the information given. Medical and communicative training must be a continuous part of the improvement strategy within the out-of-hours services, with a special focus on language and culture.

## Competing interests

The authors declare that they have no competing interests.

## Authors' contributions

EHH established the project including the data collection. EHH performed the analysis and drafted the manuscript which was re-written by SH and EHH. Both authors approved the final manuscript.
